# Application of Machine Learning Approaches for Classifying Sitting Posture Based on Force and Acceleration Sensors

**DOI:** 10.1155/2016/5978489

**Published:** 2016-10-27

**Authors:** Roland Zemp, Matteo Tanadini, Stefan Plüss, Karin Schnüriger, Navrag B. Singh, William R. Taylor, Silvio Lorenzetti

**Affiliations:** ^1^Institute for Biomechanics, ETH Zurich, Vladimir-Prelog-Weg 3, 8093 Zurich, Switzerland; ^2^Seminar for Statistics, ETH Zurich, Rämistrasse 101, 8092 Zurich, Switzerland

## Abstract

Occupational musculoskeletal disorders, particularly chronic low back pain (LBP), are ubiquitous due to prolonged static sitting or nonergonomic sitting positions. Therefore, the aim of this study was to develop an instrumented chair with force and acceleration sensors to determine the accuracy of automatically identifying the user's sitting position by applying five different machine learning methods (Support Vector Machines, Multinomial Regression, Boosting, Neural Networks, and Random Forest). Forty-one subjects were requested to sit four times in seven different prescribed sitting positions (total 1148 samples). Sixteen force sensor values and the backrest angle were used as the explanatory variables (features) for the classification. The different classification methods were compared by means of a Leave-One-Out cross-validation approach. The best performance was achieved using the Random Forest classification algorithm, producing a mean classification accuracy of 90.9% for subjects with which the algorithm was not familiar. The classification accuracy varied between 81% and 98% for the seven different sitting positions. The present study showed the possibility of accurately classifying different sitting positions by means of the introduced instrumented office chair combined with machine learning analyses. The use of such novel approaches for the accurate assessment of chair usage could offer insights into the relationships between sitting position, sitting behaviour, and the occurrence of musculoskeletal disorders.

## 1. Introduction

Nowadays, approximately 75% of all employees in industrial countries have jobs that require working in a seated position [1], resulting in prolonged spinal postures that are thought to be associated with an increased risk of musculoskeletal disorders in the back, neck, shoulders, arms, and legs [2, 3]. Chronic low back pain (LBP) is one such musculoskeletal disorder that is commonplace, with almost everyone experiencing it at one time or the other [4]. Prolonged periods of sitting hinder or restrict the movement of the lower spine and, therefore, prevent changes in the hydrostatic pressure in the intervertebral discs and seem to be detrimental for nutrition of the vertebral disc [5–7]. Krämer [8] reported that periodic changes in the loads of the intervertebral discs are critically important for not only their nutrition but also resistance against pathological changes, since frequent changes between high and low disc loads are able to induce an effective pump mechanism in the vertebral discs [9]. Studies have shown that lumbar disc pressure is strongly dependent on the sitting position [10, 11]. These authors also highlighted the difference in muscular activity in the back between the different sitting positions and the different inclination angles of the backrest [10]. Furthermore, while changing position from an upright to a reclined as well as to a forward inclined sitting position, the chair user can adapt the position of the intervertebral discs [12, 13]. Thus, most chair manufacturers now provide sitting facilities that incorporate a variety of sitting positions. However, the relationship between the sitting behaviour (e.g., change frequency, number of seating positions, time in each specific sitting position, etc.) and the occurrence of LBP is not well understood [6]. Furthermore, office workers often use only a few potential sitting positions and it is unusual for most people to check their sitting behaviour and posture during concentrated working [14–16]. Additionally, sitting behaviour can also contain information about the actual level of comfort or discomfort of the chair user [17]. Therefore, it would be of great benefit to have an instrumented office chair that is able to make the user aware of their sitting position as well as provide feedback in cases of excessive periods in the same working position. This idea led us to the development of an instrumented office chair with acceleration and pressure sensors integrated into the backrest and seat pan cushions as well as the armrests in order to analyse sitting behaviour.

The concept of instrumented or sensing chairs was first introduced by Tan and coworkers in 1997 [18–20]. The authors placed surface-mounted pressure distribution sensor mats over the seat pan as well as over the backrest to obtain real-time information of the chair-user interaction. Within the project the authors addressed different possible long-term goals such as the possibility of directly controlling a video camera in a remote conference room, of gathering sitting position information to give a feedback to the user, or of helping furniture designers evaluate their new chairs by observing sitting behaviour over a certain period of time. Beyond these potential applications in the office environment, it is also conceivable to monitor the pressure distribution on wheelchairs or driver's or passenger's seats to automatically adjust or redistribute the pressure distribution on the chair surface so as to improve seating comfort, to adjust the airbag force in case of an accident, to provide navigation guidance, or to make the driver aware of the traffic situation in order to prevent accidents.

In previous studies, the use of pressure sensor mats on the seat pan and on the backrest has allowed the detection of ten different static sitting positions with an overall classification accuracy of 79% for subjects with which the pattern learning system is not familiar [19]. Mota and Picard [21] used the same measuring system but in a dynamic setup to analyse nine different sitting positions. In order to teach the pattern recognition algorithm, two observers labelled the different sitting positions by means of video analysis, producing an overall classification accuracy of 87.6% in new subjects. In a similar manner, Meyer and coworkers [22] used a textile pressure sensor mat with 96 elements on the seat pan and one element on the backrest in order to classify 16 different static sitting positions. By applying the 16 best features, selected by the sequential forward selection algorithm [23], to a Naïve Bayes classifier the authors reported a classification accuracy of 82%. The* Smart Cushion* system [24] introduced by Xu and coworkers consists of a 16 × 16 textile pressure sensor mat placed at the seat pan of a conventional chair. By firstly converting the 2D pressure map into a 1D pressure profile sequence and applying a time warping-based classification algorithm, an accuracy of almost 86% was achieved for seven different postures. These studies [19, 21, 22, 24] demonstrate that it is possible to detect different sitting positions in both static and dynamic setups with considerable accuracy by means of conventional as well as textile pressure sensor mats.

In order to reduce the complexity as well as the cost of the measurement system, some studies aimed to analyse the sitting position using several single axis force or pressure sensors. For example, Mutlu and coworkers [25] evaluated the pressure distribution data of Tan and coworkers [19] in order to place 19 pressure sensors on the seat pan and the backrest of an office chair as close as possible to the optimal position for the recognition of the same ten different sitting positions. By means of the* SimpleLogistic* classifier from the Weka library [26] they achieved a mean classification accuracy of 78%. Daian and coworkers [15] placed one force sensor on the seat pan and one on the backrest to detect if somebody is sitting. With the use of this system, the authors were able to determine (a) whether the chair had been used or not, (b) whether the person was sitting in an adequate position, (c) or whether the person was sitting in an inadequate position. However, the sensor system was not validated and therefore it is difficult to assess the accuracy of the classification approach. In a similar manner, instrumenting the seat pan of a conventional office chair with four force sensors located at each corner under the seating surface has allowed the classification of eight different sitting postures [16]. As in the previous case, however, this study did not provide the accuracy of the classification approach and it is therefore difficult to interpret the quality of the reported seating posture classification. Hu and coworkers [27] presented their* PoSeat*, a smart seat cushion with the aim of preventing chronic back pain. In order to keep the costs low, they experimented with multiple placement schemes with 16 pressure sensors. Finally, the authors came up with a solution using six pressure sensors (four at the backrest and two at the seat pan) and one accelerometer within the cushion covering the backrest. By using Support Vector Machines the* PoSeat* was able to detect five different sitting positions with an unspecified classification accuracy.

To our knowledge, there is no other study that has analysed sitting behaviour by means of classifying the sitting positions using force or pressure sensors. Therefore, there is a lack of knowledge about the possibility of classifying sitting behaviour by means of integrated pressure sensors in office chairs. Nevertheless, studies have shown that feedback devices (graphical, physical, or vibrotactile interruption feedback) have an enormous potential to motivate chair users to improve their sitting behaviour [16], to make chair users become more aware of their sitting posture [15], and to promote the adoption of beneficial postures which can be effective in preventing workplace-related musculoskeletal disorders [28]. Therefore, the aim of this study was to develop an instrumented office chair with force and acceleration sensors in order to assess the frequency of different sitting positions in office chairs. Moreover, different pattern recognition algorithms were applied and their performances compared by analysing the mean and the subject-specific and the position-specific classification accuracy. Finally an assessment of sensor importance was performed in order to rate the requirement for each sensor and location.

## 2. Materials and Methods

### 2.1. Measuring System and Subjects

In order to analyse sitting behaviour of subjects during office work, three conventional office chairs (ID® chair, Vitra AG) were equipped with a custom built Motion-Module (accelerometer, gyroscope, and magnetometer; MPU-9250 Nine-Axis, MEMS MotionTracking™ Devices, InvenSense, California, USA) attached to the rear of the backrest to assess global chair movement and backrest angle of the office chair (Figure A1, in Supplementary Material available online at http://dx.doi.org/10.1155/2016/5978489). Furthermore, ten pressure sensors were fixed within the seat pan, four were fixed on the backrest, and three were fixed on each armrest. The latter three sensors were connected in series; hence the equipped office chair provided 16 different pressure sensor signals. The pressure sensors (FSR® 406, Interlink Electronics; 43.69 mm square sensor, thickness: 0.45 mm) changed their resistance as a function of the loading. The sensors were attached using a double-sided adhesive tape onto the chair padding and the plastic armrests and additionally fixed with tape at the soldering joint in order to protect the most vulnerable area of the sensors. The fabric cover of the office chair was then pulled over the sensors. Due to the fact that the pressure sensors are highly sensitive to the contact area (material properties and geometry), all sensors were calibrated by means of a wooden stamp loaded with several weights (0.6 kg, 1.1 kg, 1.7 kg, 3 kg, and 4.3 kg). The calibration procedure was repeated three times for every sensor and a linear regression equation was fitted through the measured sensor values in relation to the applied forces (similar to the calibration procedure of [29]). This calibration enabled us to make the sensor data of the three office chairs comparable.

Forty-one healthy subjects (16 females and 25 males) with an average age of 38 years (range: 24–64 y), a mean height of 177 cm (range: 160–200 cm), and an average weight of 77 kg (range: 53–126 kg) gave informed consent to participate in this study which was approved by the ethics committee of the ETH Zurich (number EK 2013-N-03). A wide range of subject age, height, and weight was selected in order to find an algorithm suitable to detect the sitting position for a broad population.

### 2.2. Measurements

The instrumented office chair setup presented here allowed the force distribution patterns as well as the backrest angle of different sitting positions to be analysed. Here, all 41 subjects were requested to sit successively in the seven most common predefined sitting positions [16, 21, 24] one after another (Supplementary Materials: Figure A2). This procedure was repeated four times. In order to familiarise the subjects to the different sitting positions a hand-out with the illustrations was provided (Supplementary Materials: Figure A2). As we wanted to capture the natural variation in seating positions, the experimenters did not provide additional instructions or any feedback to the subjects. Participants were asked to sit in each of the seating positions for at least five seconds. The force and Motion-Module data were recorded at 100 Hz for one second. To do so, the examiner manually started the one-second measurement after the subjects had been sitting in a particular position for approximately three seconds. Between each measurement, subjects were asked to stand up in order to reset the pressure sensors as well as to avoid any influence of the previous sitting positions on the following one.

### 2.3. Data Set and Features

Data analysis was performed using MATLAB (vR2013a, MathWorks Inc., Natick, USA). The backrest angle was determined for every measurement frame by assessing the intermediate angle of the gravitational force vector of the unloaded chair and the same vector of the sitting position measurement using the 3D acceleration data of the Motion-Module. The input data for the classification paradigm included the median of one-second duration of the force data divided by the subject's body weight as well as the median of one-second duration of the calculated backrest angles resulting in a total of 17 different explanatory variables. Here, median values were extracted in order to ensure that all the 17 input data vectors were robust against outliers resulting from sensor fluctuations (which in retrospect never occurred).

### 2.4. Classification and Validation

In order to quantify the reliability of the different classification algorithms a Leave-One-Out (LOO) cross-validation was performed where the data of all subjects except one was used as training data and the remaining subject was used for validation. The predicted position was then identified as either correctly or wrongly classified. The fraction of the misclassified observations was defined as the LOO estimate for a particular subject. This was repeated for all subjects and the mean value of the LOO estimate of the 41 subjects was calculated, which can be considered as approximately $O\left(1/\sqrt{n}\right)$ of the true error [30–33], where *O*(·) stands for the Bachmann-Landau notation [34, 35] and *n* is equal to the number of subjects. The classification accuracy of a particular classification method was then defined as one minus the LOO estimate. The estimate of the LOO procedure is therefore almost unbiased and closely represents the true generalisation error [36, 37]. Due to the fact that LOO estimates the generalisation error of a model trained with *n* − 1 subjects, it provides a lower classification performance than the real model with *n* subjects [38].

Studies have shown that a lot of different algorithms can be used for the classification of sitting positions with satisfactory accuracy ranging from 78% to 88% [19, 21, 22, 24, 25]. Since the performance of a classification algorithm is highly dependent on the used data set, we compared the five algorithms (as well as their combinations) that are generally thought to function best for classification problems [39–41]. Unless specified, MATLAB's (vR2013a, MathWorks Inc., Natick, USA) default parameter values were used for all pattern recognition algorithms.

*Classification Paradigms*

*(1) Support Vector Machines (SVMs) [42, 43]*. SVMs with a linear, quadratic, 3rd, 4th, 5th, and 6th polynomial order kernel function and with a Gaussian Radial Basis Function (RBF) kernel were used to classify the different sitting positions. The scaling factor *σ*_RBF_ of the RBF kernel was varied between 0.01 and 4.00 in 0.01 steps. Sequential Minimal Optimisation (SMO) was used to find the separating hyperplane and the maximum number of iterations of the main loop was set at 10^6^. 

*(2) Multinomial Regression (MNR) [39]*. Each sitting position measurement was predicted to belong to the sitting position associated with the highest probability of the multinomial Regression model for nominal responses.

*(3) Boosting [44]*. For all Boosting methods the number of ensemble learning cycles was set at 500. For the Boosting method* Subspace*, the weak learners* Discriminant* as well as* kNN* (*k*-Nearest-Neighbour) were applied. All other Boosting methods (*AdaBoostM2*,* LPBoost*,* TotalBoost*,* RUSBoost,* and* Bag*) were trained with* Discriminant* and* tree* weak learners.

*(4) Neural Networks (NNs) [45]*. A conventional feedforward neural network (*feedforwardnet*) and various modifications (such as* fitnet*,* patternnet*, and* cascadeforwardnet*) with five different training functions were used. The network training functions were Levenberg-Marquardt backpropagation (*trainlm*), scaled conjugate gradient backpropagation (*trainscg*), gradient descent backpropagation (*traingd*), and gradient descent with momentum backpropagation (*traingdm*) as well as gradient descent with momentum and adaptive learning rate backpropagation (*traingdx*). The hidden layer size was varied between five and 25 for all feedforward networks. Furthermore also Radial Basis Networks, particularly an exact Radial Basis Network (*newrbe*), a generalised regression neural network (*newgrnn*), and probabilistic neural network (*newpnn*), were designed. The scaling factor *σ*_RBF_ of the radial basis functions was varied between 0.01 and 4.00 in steps of 0.01.

*(5) Random Forest (RF) [41]*. An ensemble of 500 bagged decision trees was created with a number of variables to select at random for each decision split (*NVarToSample*) between two and five.

*(6) Combination of Boosting, NN, and RF*. In order to improve the classification algorithm, the three best performing approaches were combined with each other. The mean value of the class membership probabilities of the* Bag* Boosting trained with* tree* weak learners, the conventional feedforward neural network (*feedforwardnet*) using Levenberg-Marquardt backpropagation (*trainlm*) with a hidden layer size of 22, and the RF method with four variables to select at random for each decision split was calculated and the data were assigned to the sitting position with the highest probability.

The importance of the 17 different explanatory variables (16 pressure sensors and backrest angle) was analysed by performing the out-of-bag feature importance evaluations (*OOBPermutedVarDeltaError*) of the RF method [46] with the best performing parameters (*NVarToSample* = 4). Furthermore, by using the results of the feature importance analysis, we performed the LOO cross-validation (RF,* NVarToSample* = 4) with different explanatory variable combinations in order to analyse the importance of the backrest angle (scenarios A, C, and E) as a predictor, to evaluate the possibility of reducing some force channels (scenarios B, D), and to determine whether the force sensors on the seat pan were sufficient for classifying the different sitting positions (scenario F). The six different analysed scenarios were as follows (Figure 5):

 A: all channels except the backrest angle (1–16).
 B: all channels except the ones at the lower backrest (7/15).
 C: all channels except the backrest angle and the sensors at the lower backrest (7/15).
 D: only the pressure sensor channels 2, 6, 10, and 14, as well as backrest angle 17.
 E: only the pressure sensor channels 2, 6, 10, and 14.
 F: only pressure sensor channels of the seat pan (1–5, 9–13).

## 3. Results

### 3.1. Sitting Posture Classification and Validation

**Figure 1:**
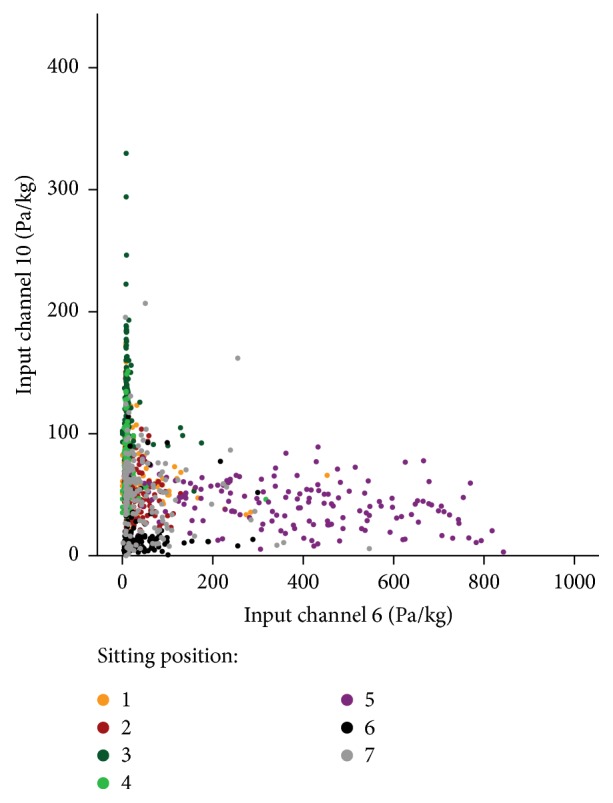
Pressure values of input channel 6 against the values of input channel 10 for the seven different sitting positions (as shown in Figure A2 (Supplementary Materials); 1: upright position, 2: reclined position, 3: forward inclined position, 4/5: laterally tilted right/left position, 6/7: crossed legs, the left leg over the right one/the right leg over the left one).

A scatterplot of all pressure values of input channel 6 against input channel 10, exemplarily representing one of the pairs of sensors with the highest optical distinction power for the differentiation of the seven sitting positions, is shown in Figure 1. It was almost impossible to distinguish any sitting position from another due to the high complexity as well as variability within our data set. The classification accuracy for the different pattern recognition algorithms (Table 1) varied between 25.3% (NN,* patternnet*, and* traingd(24)*) and 90.9% (RF,* NVarToSample* = 4). By tuning the parameters of the five different pattern recognition methods, a classification accuracy of at least 82.7% could be achieved for every method. The Boosting (*Bag*,* tree*), NN (*feedforward*,* trainlm(22)*), and RF (*NVarToSample* = 4) showed the three best recognition performances with accuracies of 90.4%, 90.4%, and 90.9%, respectively. Finally, further improvement in the classification accuracy was not observed even after combining the three most successful pattern recognition paradigms.

**Figure 2:**
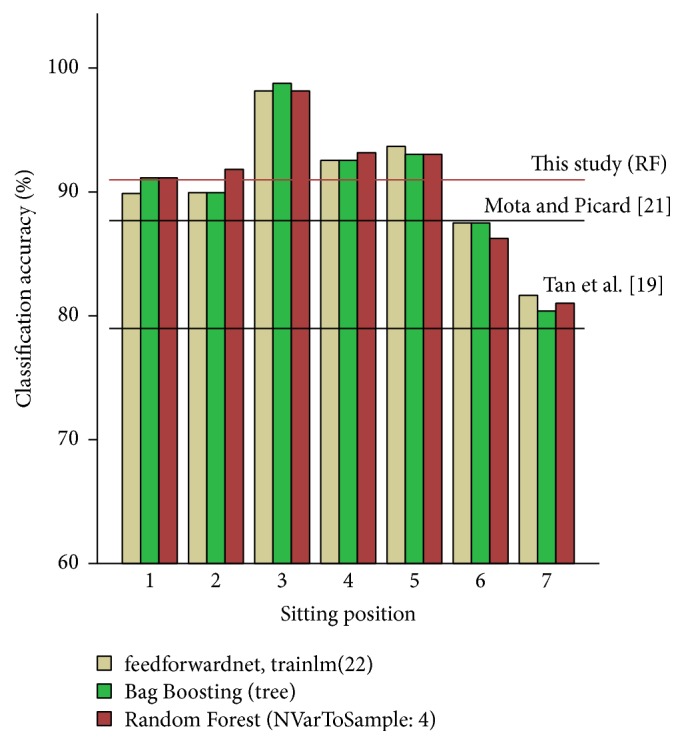
Classification accuracy of the seven different sitting positions (as shown in Figure A2 (Supplementary Materials); 1: upright position, 2: reclined position, 3: forward inclined position, 4/5: laterally tilted right/left position, and 6/7: crossed legs, the left leg over the right one/the right leg over the left one) for the three best performing classification methods (NN (beige), Boosting (green), and RF algorithm (red)), each using the parameters leading to the highest classification accuracy. The horizontal lines represent the classification accuracy of Tan et al. [19] and Mota and Picard [21] (black) as well as that of the current study using RF (red).

The classification accuracy was then further categorised into the seven different sitting positions for the three most successful classification methods (Figure 2). The accuracies clearly varied according to the sitting position with the worst classified sitting postures being the crossed leg positions with approximately 81% accuracy, while the posture with forward inclination was classified most accurately at about 98% for all of the three methods.

**Figure 3:**
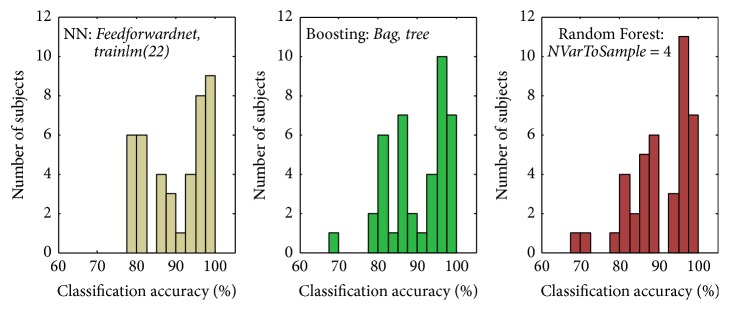
Histogram of the classification accuracy of the 41 different subjects for the three best classification methods (NN, Boosting, and RF), each using the parameters leading to the highest classification accuracy.

The histograms of the classification accuracy of the different subjects (Figure 3) were very similar for the Boosting (*Bag*,* tree*) and the RF (*NVarToSample* = 4). Both the Boosting (*Bag*,* tree*) and the RF (*NVarToSample *= 4) method showed the lowest accuracy value of 67.9% for one subject, whereas for the NN (*feedforward*,* trainlm(22)*) the lowest classification accuracy was 77.8%.

### 3.2. Random Forest Feature Importance

**Figure 4:**
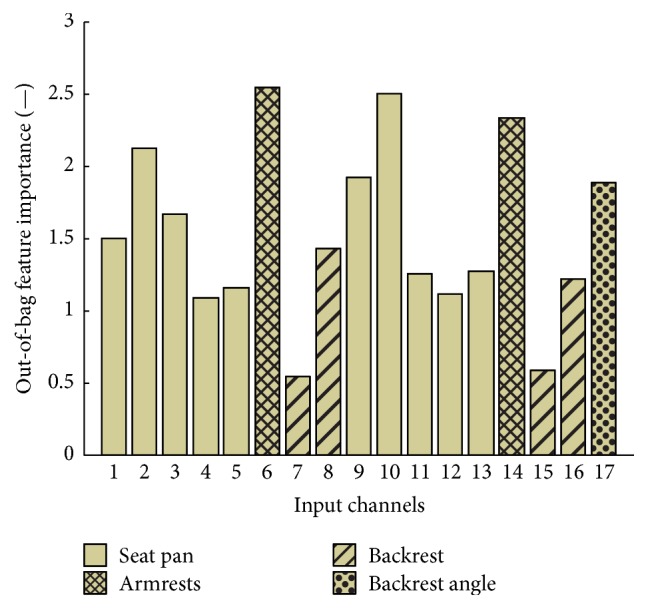
Out-of-bag feature importance of the 17 explanatory variables: pressure sensors (1–16, Supplementary Materials: Figure A1) and backrest angle (17) for the RF (*NVarToSample* = 4). The sensors on the seat pan are presented in a plain colour, those on the armrests are presented as crosshatched ones, those on the backrest are presented as normal hatched ones, and the backrest angle is in patterned dots.

The mean value of the out-of-bag feature importance of the 17 predictors was around 1.5 (Figure 4). The highest values were found for the sensors at the armrests (6, 14; Supplementary Materials: Figure A1) as well as the two sensors in the anterior part of the seat pan (2, 10; Supplementary Materials: Figure A1) with values between 2.1 and 2.5, whereas the lowest importance rating could be found for the two sensors at the lower backrest (7, 15; Supplementary Materials: Figure A1) with values lower than 0.6.

**Figure 5:**
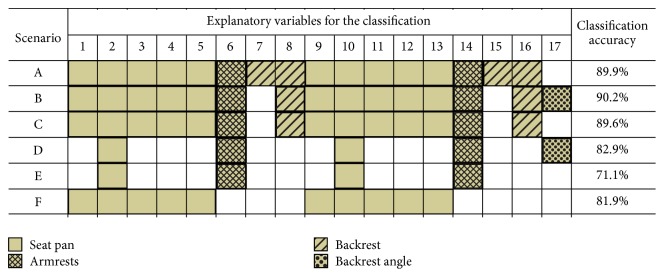
Classification accuracy for different explanatory variable combinations (scenarios A–F) using RF (*NVarToSample* = 4): columns 1–16 represented the pressure sensors (Supplementary Materials: Figure A1) and column 17 represented the backrest angle. The sensors on the seat pan are presented in a plain colour, those on the armrests are presented as crosshatched ones, those on the backrest are presented as normal hatched ones, and the backrest angle is in patterned dots.

By neglecting the backrest angle, as well as the two sensors of the lower backrest, a classification accuracy of 89.6% was determined (Figure 5). By only considering the four sensors with the highest feature importance value (2, 6, 10, and 14; Supplementary Materials: Figure A1), an accuracy of 71.1% was determined. Furthermore, using only the sensors on the seat pan showed a classification accuracy of 81.9%.

## 4. Discussion

Despite increasingly prolonged seating periods required for office jobs, the relationship between the sitting behaviour and the occurrence of LBP is hardly understood [6]. One challenging aspect of addressing this issue is to correctly classify sitting position in office environments and subsequently apply this classification for quantifying sitting behaviour. The aim of our study was therefore to develop an instrumented office chair in order to automatically classify various sitting postures as an essential first step towards assessing sitting behaviour for chair users. Here, with the application of 16 pressure sensors, as well as a Motion-Module for assessing back rest angle and chair motion, we were able to detect seven different sitting positions with a classification accuracy of over 90% (RF,* NVarToSample* = 4). The use of Leave-One-Out analyses for assessing new subjects that were entirely unfamiliar with the classification algorithm ensured that this validation accuracy was not only conservative but also realistic for application in new environments.

The complexity of our data set was too high in order to classify the different sitting positions by means of simple threshold based algorithms (Figure 1). The use of advanced algorithms such as SVMs, MNR, Boosting methods, and NNs as well as RFs with different algorithm parameters was therefore necessary for classifying the different sitting positions. The performance of the five different classification methods was very similar when tuning the algorithm's specific parameters; however only three methods, Boosting (*Bag*,* tree*), NN (*feedforward*,* trainlm(22)*), and RF (*NVarToSample* = 4), showed a classification accuracy above 90%. The results achieved in our study present an improvement over the values reported in similar studies [19, 21, 22, 24, 25], which generally ranged between 78% and 88%. The studies from Tan and coworkers [19] as well as Mota and Picard [21] used an instrumented chair with pressure sensor mats on the seat pan and the backrest. Therefore, these studies used some 4032 sensor units compared to the simpler 17-sensor system (16 pressure sensors and one Motion-Module) considered here. Our improved classification accuracy, despite reduced levels of information, is likely to be due to the effective utilisation of modern algorithms [39, 40] and improved spatial placement of the sensors around the seat pan and backrest. The current study also shows that the sensors on the lower backrest (7, 15; Supplementary Materials: Figure A1) as well as the Motion-Module can be left out without any notable loss in the overall classification accuracy. Furthermore, it is still possible to assess the different sitting positions by only using the pressure sensors on the seat pan with reasonable classification accuracy (81.9%).

Dreischarf et al. [47] highlighted the importance of supporting the upper body using the arms in order to decrease spinal loads. By means of our instrumented office chair it is possible not only to accurately detect the users' current sitting position but also to quantify the armrest usage, which could play an important role in understanding and preventing LBP in the office environment.

By looking at the different sitting positions individually, we found the highest classification accuracies for the forward inclined sitting position (3; Supplementary Materials: Figure A2). This is plausible inasmuch as the forward inclined sitting position shows differences in almost every sensor value compared to all other positions. Here, an increase in the anterior and decrease in the posterior seat pan sensors are accompanied by no contact to the backrest, since the back is entirely unsupported, with more weight probably supported by the legs. Furthermore, this is the only position with a forward inclined backrest angle. The lowest classification accuracies were determined for the two sitting positions with crossed legs (6, 7; Supplementary Materials: Figure A2). Surprisingly, despite the symmetry of the two sitting positions with crossed legs, the classification accuracy differed by approximately 6%. This might be explained by the fact that many of the analysed subjects reported a preference to have the left leg over the right one in the crossed leg sitting positions, which is likely a result of the fact that the limb dominance was not evenly distributed across the analysed subjects. As a result, the variability in the performance of the crossed legged sitting position with the right leg over the left one was likely to be higher and therefore could have led to a lower classification accuracy compared to the sitting position with the left leg over the right one. Importantly, in daily office work, where subjects are not asked to sit in predefined positions, workers are unlikely to sit in less preferred positions and therefore the general classification accuracy of the techniques used here would be even higher. Furthermore, in a real working environment computer mouse usage can also result in an asymmetric sitting as well as upper body positions. However, the role of any such asymmetrical sitting bias and its possible role in low back pain remain to be elucidated.

It is important to note that our measuring system only allows classification of the seven predefined postures with an accuracy of over 90%. Hence, if a subject is sitting in another (unknown) position, the algorithm would predict the nearest known sitting position that shows the most similar input channel pattern. Furthermore, it must be kept in mind that some pattern recognition algorithms were given a stronger weight due to the fact that some methods were modified with many different parameter values (e.g., NN) and others were not (e.g., MNR). However, any such weighting could be considered of only low importance, since the aim of our study was not to compare different classification algorithms in general. Our strategy rather enabled us to find the classification algorithm and parameters performing the best for our data set. Nevertheless, the RF method showed the best performance for our data set, even though we adapted only one single parameter of the algorithm that resulted in four different RF executions. Furthermore, in addition to the fact that this method showed the highest accuracy, the authors would recommend the use of RF since this method is one of the fastest algorithms, it provides direct estimates of the variable importance, and the algorithm does not overfit [41].

With 41 subjects in 7 different sitting positions, each 4 times, the total sample size of 1148 sitting assessments could be considered to be rather high—and, indeed, this was confirmed by the excellent performance of the classifiers, which provided appropriate confirmation that the training set was more than sufficient to provide a baseline understanding for classifying sitting positions. However, should assessment of a greater number of sitting positions be required, further data sets would be beneficial to ensure similar classification performance. In future studies, the introduced instrumented office chair could be used to analyse sitting behaviour in the office environment in order to gain a better understanding of the relationship between the amount of time spent in a sitting position and the occurrence of LBP.

## 5. Conclusions

To summarise, the present study demonstrated the ability of sensor technology, together with machine learning analyses, to accurately classify different occupational sitting positions. The use of such novel approaches for the accurate assessment of chair usage could offer insights into the relationships between sitting position, sitting behaviour, and the occurrence of musculoskeletal disorders but could also provide important knowledge in other fields, for example, sleeping disorders, or in subjects suffering clinical pathologies such as spinal cord injuries. In the future office environment, sitting assessment technologies could become commonplace in order to help to raise user's awareness of their sitting behaviour and may help towards reducing the occurrence of LBP.

## Supplementary Material

The supplementary material includes the experimental set up, including the sensor, sensor distribution and the instrumented chair as well as the different sitting positions.

## Figures and Tables

**Table 1:** Classification accuracy of the different pattern recognition algorithms.

Classification algorithm	Classification accuracy
*Support Vector Machines (SVMs)*	
Linear kernel function	70.1%
Quadratic kernel function	78.5%
3rd polynomial kernel function	77.7%
4th polynomial kernel function	79.8%
5th polynomial kernel function	75.9%
6th polynomial kernel function	72.2%
RBF kernel (*σ*_RBF_ = 2.53)	**82.7%**

*Multinomial Regression (MNR)*	**87.8%**

*Boosting*	
*Subspace*	
*kNN* ^a^	47.6%
*Discriminant* ^a^	72.4%
*AdaBoostM2*	
*Discriminant* ^a^	85.2%
*Tree* ^a^	78.8%
*LPBoost*	
*Discriminant* ^a^	82.1%
*Tree* ^a^	62.3%
*TotalBoost*	
*Discriminant* ^a^	75.1%
*Tree* ^a^	68.5%
*RUSBoost*	
*Discriminant* ^a^	82.0%
*Tree* ^a^	27.4%
*Bag*	
*Discriminant* ^a^	81.8%
*Tree* ^a^	**90.4%**

*Neural Networks (NN)*	
Feedforward NN	
*Feedeforwardnet*	
*trainlm(22)* ^b^	**90.4%**
*trainscg(23)* ^b^	88.3%
*traingd(22)* ^b^	39.1%
*traingdm(22)* ^b^	37.2%
*traingdx(23)* ^b^	77.5%
*Patternnet*	
*trainlm(17)* ^b^	89.2%
*trainscg(15)* ^b^	85.1%
*traingd(24)* ^b^	25.3%
*traingdm(25)* ^b^	27.6%
*traingdx(18)* ^b^	71.7%
*Fitnet*	
*trainlm(21)* ^b^	89.8%
*trainscg(18)* ^b^	88.4%
*traingd(25)* ^b^	40.0%
*traingdm(24)* ^b^	36.3%
*traingdx(24)* ^b^	78.4%
*Cascade-forwardnet*	
*trainlm(22)* ^b^	90.0%
*trainscg(22)* ^b^	78.7%
*traingd(23)* ^b^	45.2%
*traingdm(21)* ^b^	39.3%
*traingdx(22)* ^b^	41.7%
Radial Basis NN	
*newrbe *(*σ*_RBF_ = 0.51)^c^	72.5%
*newgrnn *(*σ*_RBF_ = 0.08)^c^	69.5%
*newpnn *(*σ*_RBF_ = 0.08)^c^	69.5%

*Random Forest (RF)*	
*NVarToSample *= 2	90.5%
*NVarToSample *= 3	90.7%
*NVarToSample *= 4	**90.9%**
*NVarToSample *= 5	90.2%

*Combination: Boosting, NN, RF*	**90.8%**

Only the results with the highest classification accuracy for the different hidden layer sizes (NN) as well as for the different scaling factors *σ*_RBF_ (SMV with RBF kernel, Radial Basis NN) are listed. The highest accuracies are marked in bold for every category.

^a^Weak learner for the corresponding Boosting method.

^b^Neural network training function with the hidden layer size in brackets that showed the highest classification accuracy.

^c^The highest classification accuracy shown throughout variation of the scaling factor of the radial basis function *σ*_RBF_.

## References

[1] Reinecke S. M., Hazard R. G., Coleman K., Pope M. H., Kumar S. (2002). A continuous passive lumbar motion device to relieve back pain in prolonged sitting. *Advances in Industrial Ergonomics and Safety IV*.

[2] Winkel J., Jorgensen K. (1986). Evaluation of foot swelling and lower-limb temperatures in relation to leg activity during long-term seated office work. *Ergonomics*.

[3] Naqvi S. A. (1994). Study of forward sloping seats for VDT workstations. *Journal of Human Ergology*.

[4] Hoy D., Brooks P., Blyth F., Buchbinder R. (2010). The epidemiology of low back pain. *Best Practice & Research: Clinical Rheumatology*.

[5] van Dieen J. H., de Looze M. P., Hermans V. (2001). Effects of dynamic office chairs on trunk kinematics, trunk extensor EMG and spinal shrinkage. *Ergonomics*.

[6] Fryer J. C. J., Quon J. A., Smith F. W. (2010). Magnetic resonance imaging and stadiometric assessment of the lumbar discs after sitting and chair-care decompression exercise: a pilot study. *Spine Journal*.

[7] Pynt J., Higgs J., Mackey M. (2001). Seeking the optimal posture of the seated lumbar spine. *Physiotherapy Theory and Practice*.

[8] Krämer J. (1973). *Biomechanische Veränderungen im Lumbalen Bewegungssegment*.

[9] Grandjean E., Hünting W. (1977). Ergonomics of posture—review of various problems of standing and sitting posture. *Applied Ergonomics*.

[10] Andersson B. J. G., Ortengren R. (1974). Lumbar disc pressure and myoelectric back muscle activity during sitting. II. Studies on an office chair. *Scandinavian Journal of Rehabilitation Medicine*.

[11] Wilke H.-J., Neef P., Hinz B., Seidel H., Claes L. (2001). Intradiscal pressure together with anthropometric data—a data set for the validation of models. *Clinical Biomechanics*.

[12] Baumgartner D., Zemp R., List R. (2012). The spinal curvature of three different sitting positions analysed in an open MRI scanner. *The Scientific World Journal*.

[13] Zemp R., Taylor W. R., Lorenzetti S. (2013). *In vivo* spinal posture during upright and reclined sitting in an office chair. *BioMed Research International*.

[14] Phillips S. (1999). The continuing problem of OOS in the office. *Ergonomics Australia*.

[15] Daian I., Ruiten A. M. V., Visser A., Zubic S. Sensitive chair: a force sensing chair with multimodal real-time feedback via agent.

[16] Haller M., Richter C., Brandl P., Campos P., Graham N., Jorge J., Nunes N., Palanque P., Winckler M. (2011). Finding the right way for interrupting people improving their sitting posture. *Human-Computer Interaction—INTERACT 2011*.

[17] Zemp R., Taylor W. R., Lorenzetti S. (2015). Are pressure measurements effective in the assessment of office chair comfort/discomfort? A review. *Applied Ergonomics*.

[18] Tan H. Z., Ifung L., Tentland A. The chair as a novel haptic user interface.

[19] Tan H. Z., Slivovsky L. A., Pentland A. (2001). A sensing chair using pressure distribution sensors. *IEEE/ASME Transactions on Mechatronics*.

[20] Ebert D., Tan H. (2002). *Sensing Chair as an Input Device for Human-computer Interaction*.

[21] Mota S., Picard R. W. Automated posture analysis for detecting learner's interest level.

[22] Meyer J., Arnrich B., Schumm J., Troster G. (2010). Design and modeling of a textile pressure sensor for sitting posture classification. *IEEE Sensors Journal*.

[23] Kittler J. (1978). Feature set search algorithms. *Pattern Recognition and Signal Processing*.

[24] Xu W. Y., Huang M.-C., Amini N., He L., Sarrafzadeh M. (2013). eCushion: a textile pressure sensor array design and calibration for sitting posture analysis. *IEEE Sensors Journal*.

[25] Mutlu B., Krause A., Forlizzi J., Guestrin C., Hodgins J. Robust, low-cost, non-intrusive sensing and recognition of seated postures.

[26] Witten I. H., Frank E. (2005). *Data Mining: Practical Machine Learning Tools and Techniques*.

[27] Hu Y., Stoelting A., Wang Y.-T., Zou Y., Sarrafzadeh M. (2010). Providing a cushion for wireless healthcare application development. *IEEE Potentials*.

[28] Yoo W. G., Yi C. H., Kim M. H. (2006). Effects of a proximity-sensing feedback chair on head, shoulder, and trunk postures when working at a visual display terminal. *Journal of Occupational Rehabilitation*.

[29] Zemp R., Taylor W. R., Lorenzetti S. (2016). Seat pan and backrest pressure distribution while sitting in office chairs. *Applied Ergonomics*.

[30] Kearns M., Ron D. (1999). Algorithmic stability and sanity-check bounds for leave-one-out cross-validation. *Neural Computation*.

[31] Devroye L. P., Wagner T. J. (1979). Distribution-free inequalities for the deleted and holdout error estimates. *IEEE Transactions on Information Theory*.

[32] Devroye L. P., Wagner T. J. (1979). Distribution-free performance bounds for potential function rules. *IEEE Transactions on Information Theory*.

[33] Rogers W. H., Wagner T. J. (1978). A finite sample distribution-free performance bound for local discrimination rules. *The Annals of Statistics*.

[34] Bachmann P. G. H. (1894). *Zahlentheorie: Die Analytische Zahlentheorie*.

[35] Landau E. (1909). *Handbuch der Lehre von der Verteilung der Primzahlen*.

[36] Luntz A., Brailovsky V. *On Estimation of Characters Obtained in Statistical Procedure of Recognition*.

[37] Chapelle O., Vapnik V., Bousquet O., Mukherjee S. (2002). Choosing multiple parameters for support vector machines. *Machine Learning*.

[38] Braga-Neto U., Dougherty E. (2004). Bolstered error estimation. *Pattern Recognition*.

[39] Tibshirani R., Friedman J., Hastie T., Friedman J., Tibshirani R. (2009). *The Elements of Statistical Learning*.

[40] Bishop C. M. (2006). *Pattern Recognition and Machine Learning*.

[41] Breiman L. (2001). Random forests. *Machine Learning*.

[42] Burges C. J. C. (1998). A tutorial on support vector machines for pattern recognition. *Data Mining and Knowledge Discovery*.

[43] Gao S., Zhang N., Duan G. Y., Yang Z., Ruan J. S., Zhang T. (2009). Prediction of function changes associated with single-point protein mutations using support vector machines (SVMs). *Human Mutation*.

[44] Freund Y., Schapire R., Abe N. (1999). A short introduction to boosting. *Journal of Japanese Society for Artificial Intelligence*.

[45] Ripley B. D. (1996). *Pattern Recognition and Neural Networks*.

[46] Fang Y., Gao S., Tai D., Middaugh C. R., Fang J. (2013). Identification of properties important to protein aggregation using feature selection. *BMC Bioinformatics*.

[47] Dreischarf M., Bergmann G., Wilke H.-J., Rohlmann A. (2010). Different arm positions and the shape of the thoracic spine can explain contradictory results in the literature about spinal loads for sitting and standing. *Spine*.

