# Supplementary material for: Application of Machine Learning Approaches for Classifying Sitting Posture Based on Force and Acceleration Sensors
Source: Biomed Res Int. 2016 Oct 27;2016:5978489. doi: 10.1155/2016/5978489 (PMC5102712; doi:10.1155/2016/5978489)
Supplement: Supplementary file 1 — The supplementary material includes the experimental set up, including the sensor, sensor distribution and the instrumented chair as well as the different sitting positions. [file 5978489.f1.pdf]

Supplementary Figures

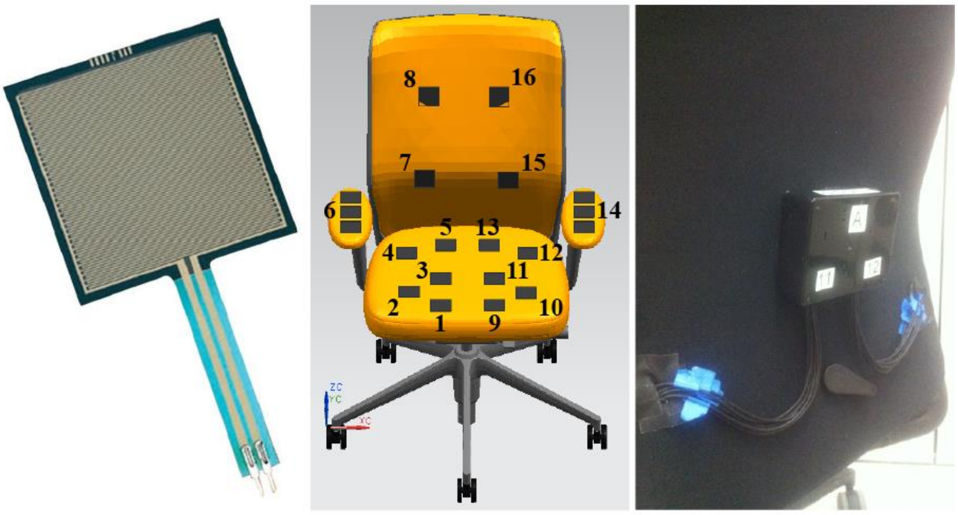

Figure A1: **Left**: A pressure sensor (FSR® 406); **middle**: Sensor distribution on the seat pan, on the backrest and on the armrests of the office chair. Sensors were numbered from one to 16; **right**: Box with a Motion-Module (accelerometer, gyroscope and magnetometer) fixed to the backrest of the office chair.

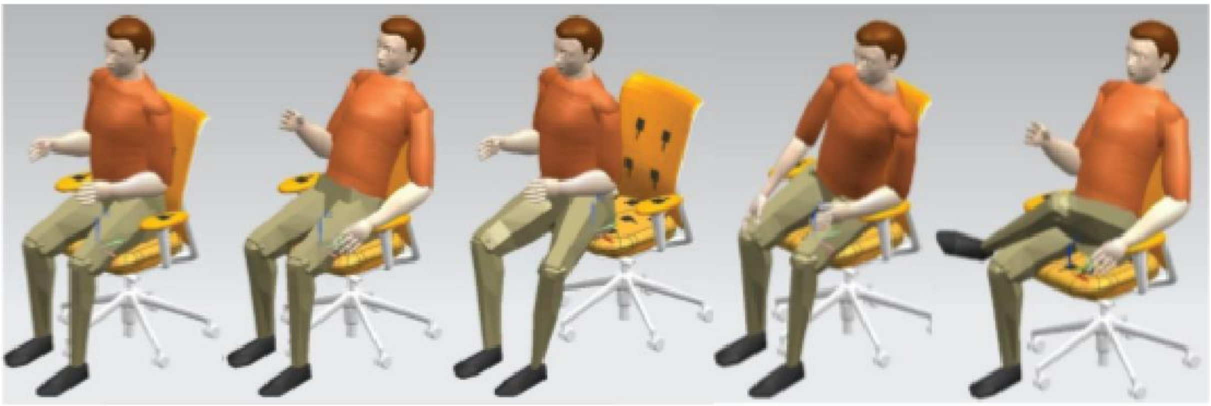

Figure A2: Sitting positions (from left to right): **1** upright, **2** reclined, **3** forward inclined, **4**/**5** laterally tilted (right/left) and **6**/**7** crossed legs (left over right/right over left).
